# DNA Damage Response Signaling Is Crucial for Effective Chikungunya Virus Replication

**DOI:** 10.1128/jvi.01334-22

**Published:** 2022-11-15

**Authors:** Sanchari Chatterjee, Sameer Kumar, Prabhudutta Mamidi, Ankita Datey, Soumya Sengupta, Chandan Mahish, Eshna Laha, Saikat De, Supriya Suman Keshry, Tapas Kumar Nayak, Soumyajit Ghosh, Sharad Singh, Bharat Bhusan Subudhi, Subhasis Chattopadhyay, Soma Chattopadhyay

**Affiliations:** a Institute of Life Sciences, Bhubaneswar, Odisha, India; b Regional Centre for Biotechnology, Faridabad, Haryana, India; c School of Biotechnology, Kalinga Institute of Industrial Technology (KIIT) University, Bhubaneswar, Odisha, India; d National Institute of Science Education and Research, an OCC of Homi Bhaba National Institute, Bhubaneswar, Odisha, India; e School of Pharmaceutical Sciences, Siksha O Anusandhan Deemed to be University, Bhubaneswar, Odisha, India; f University of Maryland, Baltimore Institute of Human Virology, Baltimore, Maryland, USA; g AIIMS, Department of Microbiology, Patrapada, Bhubaneswar, Odisha, India; h Center for Translational Medicine, Lewis Katz School of Medicine, Temple University, Philadelphia, Pennsylvania, USA; University of North Carolina at Chapel Hill

**Keywords:** chikungunya virus, DNA damage response, DDR, replication, cell cycle arrest

## Abstract

Viruses utilize a plethora of strategies to manipulate the host pathways and hijack host machineries for efficient replication. Several DNA and few RNA viruses are reported to interact with proteins involved in DNA damage responses (DDRs). As the DDR pathways have never been explored in alphaviruses, this investigation intended to understand the importance of the DDR pathways in chikungunya virus (CHIKV) infection *in vitro*, *in vivo*, and *ex vivo* models. The study revealed that CHIKV infection activated the Chk2 and Chk1 proteins associated with the DDR signaling pathways in Vero, RAW264.7, and C2C12 cells. The comet assay revealed an increase in DNA damage by 95%. Inhibition of both ATM-ATR kinases by the ATM/ATR kinase inhibitor (AAKi) showed a drastic reduction in the viral particle formation *in vitro*. Next, the treatment of CHIKV-infected C57BL/6 mice with this drug reduced the disease score substantially with a 93% decrease in the viral load. The same was observed in human peripheral blood mononuclear cell (hPBMC)-derived monocyte-macrophage populations. Additionally, silencing of Chk2 and Chk1 reduced viral progeny formation by 91.2% and 85.5%, respectively. Moreover, CHIKV-nsP2 was found to interact with Chk2 and Chk1 during CHIKV infection. Furthermore, CHIKV infection induced cell cycle arrest in G_1_ and G_2_ phases. In conclusion, this work demonstrated for the first time the mechanistic insights regarding the induction of the DDR pathways by CHIKV that might contribute to the designing of effective therapeutics for the control of this virus infection in the future.

**IMPORTANCE** Being intracellular parasites, viruses require several host cell machineries for effectively replicating their genome, along with virus-encoded enzymes. One of the strategies involves hijacking of the DDR pathways. Several DNA and few RNA viruses interact with the cellular proteins involved in the DDR pathways; however, reports regarding the involvement of Chk2 and Chk1 in alphavirus infection are limited. This is the first study to report that modulation of DDR pathways is crucial for effective CHIKV infection. It also reveals an interaction of CHIKV-nsP2 with two crucial host factors, namely, Chk2 and Chk1, for efficient viral infection. Interestingly, CHIKV infection was found to cause DNA damage and arrest the cell cycle in G_1_ and G_2_ phases for efficient viral infection. This information might facilitate the development of effective therapeutics for controlling CHIKV infection in the future.

## INTRODUCTION

Chikungunya virus (CHIKV) infection is now considered an important public health threat due to the unavailability of vaccines or antiviral treatments. In 1952, CHIKV was discovered in Tanzania, Africa, following which CHIKV has been recorded as being endemic in several countries of central Africa and southern Asia (1). The recurrence and spread of CHIKV in Asia, Europe, and the Americas have considerably escalated research on CHIKV (2–4). The symptoms include mainly high fever, nausea, back pain, rashes over the skin, myalgia, and polyarthralgia. However, it can seriously affect the well-being of the affected person for weeks, months, or even years. It belongs to the Alphavirus genus of the *Togaviridae* family and is grouped as Old World alphaviruses. The mode of transmission is by mosquito vectors, namely, Aedes aegypti and Aedes albopictus (5).

CHIKV is a single-stranded RNA virus with positive polarity. The 11.8-kb genome has an open reading frame that comprises all the nonstructural proteins (nsPs). The structural proteins are translated from a subgenomic 26S mRNA. The genome is similar to mRNA and consists of a cap and a poly A tail. nsP1 is involved in the formation of the viral cap, whereas nsP2 is a crucial protein, as it is multifunctional and is an integral component of the viral replication complex. In contrast, nsP3 is the least explored nonstructural protein, while nsP4 possesses RNA-dependent RNA polymerase activity. In addition, structural proteins, such as capsid, 6k, and envelope glycoproteins, assist in virus particle formation (6).

All the viruses utilize a plethora of strategies to manipulate the host pathways for their own survival and effective replication. One of the strategies involves the activation of the DNA damage response (DDR) pathways to utilize the DNA repair proteins. However, the mechanism underlying the induction of DNA damage signaling has not been completely elucidated so far. Several DNA and few RNA viruses interact with the cellular proteins involved in DDR, which can further lead to the upregulation or inhibition of these proteins to facilitate infection (7, 8). The DDR pathways consist of orchestrated arrays of proteins. At the core of the DDR pathways are the members of the phosphoinositide 3 (PI-3) kinase family of proteins, mainly ataxia-telangiectasia mutated kinase (ATM), which gets activated by double-strand DNA breaks, and ATM-Rad3 related kinase (ATR), which is rapidly activated in the presence of single-strand DNA breaks, leading to the phosphorylation of several downstream protein targets (9).

DNA viruses, such as papillomaviruses, appeared to activate ATM in differentiating cells, which is vital for the formation of virus replication foci (10). Viral DNA replication of human cytomegalovirus stimulates the ATM pathway, which hampers DDR by altering the localization of checkpoint proteins to the cytoplasm from the nucleus (11). Previous investigations have revealed that the herpes simplex virus (HSV) infection triggers a cellular DNA damage response, leading to the activation of ATM and abrogation of the ATR signaling pathway, which are crucial for viral replication (12, 13). In the corneal epithelium, Check point kinase-2 (Chk2) plays an important role in the HSV-1 replication (14). Furthermore, viral interferon regulatory factor 1 interacts with ATM kinase and inhibits its kinase activity in Kaposi’s sarcoma-associated herpesvirus (15). RNA viruses, such as hepatitis C virus (HCV), has been found to cause G_2_/M phase cell cycle arrest (16). Moreover, ATM and Chk2 play pivotal roles in replicating the RNA of this virus. Studies have shown that NS5B of HCV interacts with both ATM and CHK2, while its NS3-NS4A interacts with ATM (17). The Japanese encephalitis virus (JEV) reduces the proliferation of neural progenitor cells and modulates the cell cycle (18).

As the DDR pathways have never been investigated in alphaviruses, an attempt was made to determine the importance of the DDR pathways in CHIKV infection and to uncover the mechanisms via which CHIKV modulates the pathways using *in vitro*, *in vivo*, and *ex vivo* models.

## RESULTS

### Induction of DNA damage signaling pathways following CHIKV infection.

**FIG 1:**
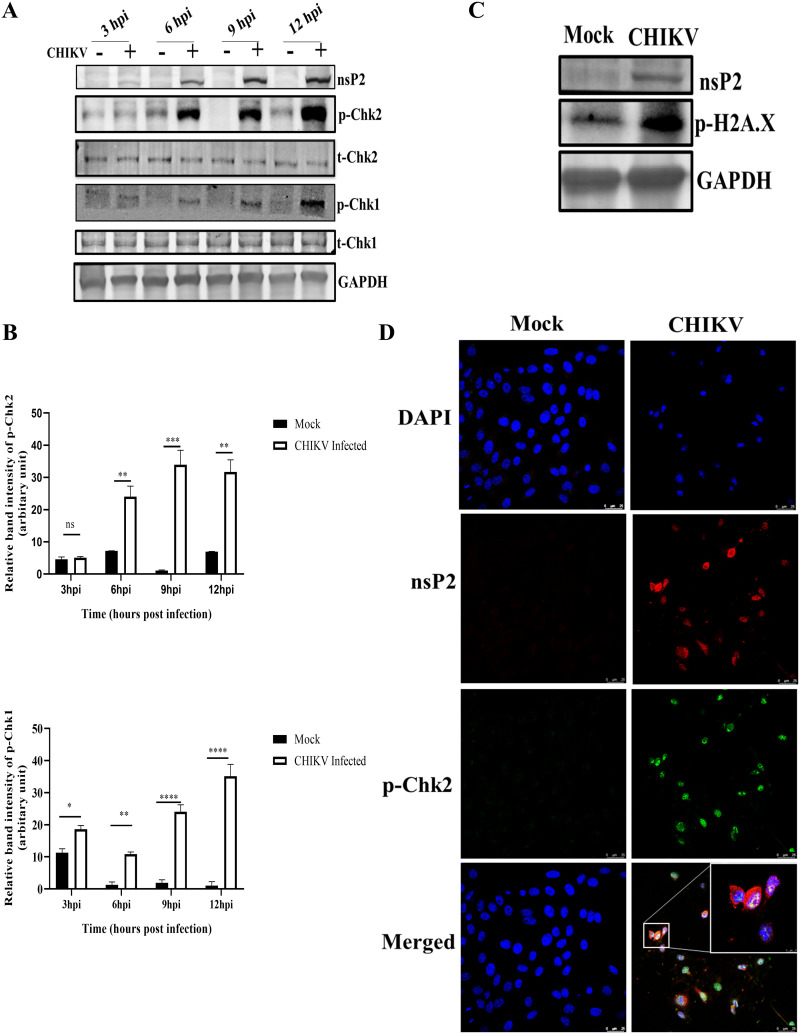
CHIKV infection induces DDR pathways. The Vero cells were mock or CHIKV infected and harvested at various time points. (A) Western blotting was performed using the nsP2, p-Chk2 (Thr-68), total Chk2, p-Chk1(Ser354), total Chk1, and GAPDH antibodies. (B) Bar diagrams showing relative band intensities of p-Chk2 and p-Chk1 at different time postinfection. Data of three independent experiments are shown as mean ± SD. (C) Western blot depicting the level of p-H2A.X (Ser-139) in mock and infected samples. (D) Mock or infected Vero cells were stained with the p-Chk2 and nsP2 antibodies. Nuclei were counterstained with DAPI. Scale bar = 25 μm. *, *P* ≤ 0.05; **, *P* ≤ 0.01; ***, *P* ≤ 0.001; and ****, *P* ≤ 0.0001 were considered statistically significant. ns, not significant.

Chk2 and Chk1 are protein kinases acting downstream as mediator proteins of the ATM and ATR arms of the DDR pathway (9). To investigate whether this pathway is activated during CHIKV infection, the phosphorylation status of Chk2 and Chk1 in CHIKV-infected cells were examined. First, Vero cells were infected with CHIKV with a multiplicity of infection (MOI) of 2 and harvested at different time points. The protein levels were evaluated using Western blot analysis. It was found that Chk2 and Chk1 were phosphorylated in the CHIKV-infected cells (Fig. 1A and B). Interestingly, the levels of phosphorylation of both Chk2 and Chk1 were increased gradually with time in CHIKV-infected cells compared with those in the corresponding mock cells. The total Chk2 and Chk1 protein levels remained unaltered in every time point as shown in Fig. 1A. Furthermore, phosphorylation of the γH2A.X was assessed to confirm the induction of DDR by CHIKV. The Vero cells were infected with CHIKV and processed for Western blot analysis. Results showed that γH2A.X was phosphorylated after CHIKV infection compared with that observed in the mock cells (Fig. 1C). To further confirm the phosphorylation of p-Chk2 following CHIKV infection, immunofluorescence analyses were performed and p-Chk2 was found to colocalize with nsP2 of CHIKV (Pearson’s coefficient *r* = 0.632) (Fig. 1D). Together, these findings indicate that the DDR pathways are activated following CHIKV infection.

### DNA damage is enhanced in CHIKV-infected cells.

**FIG 2:**
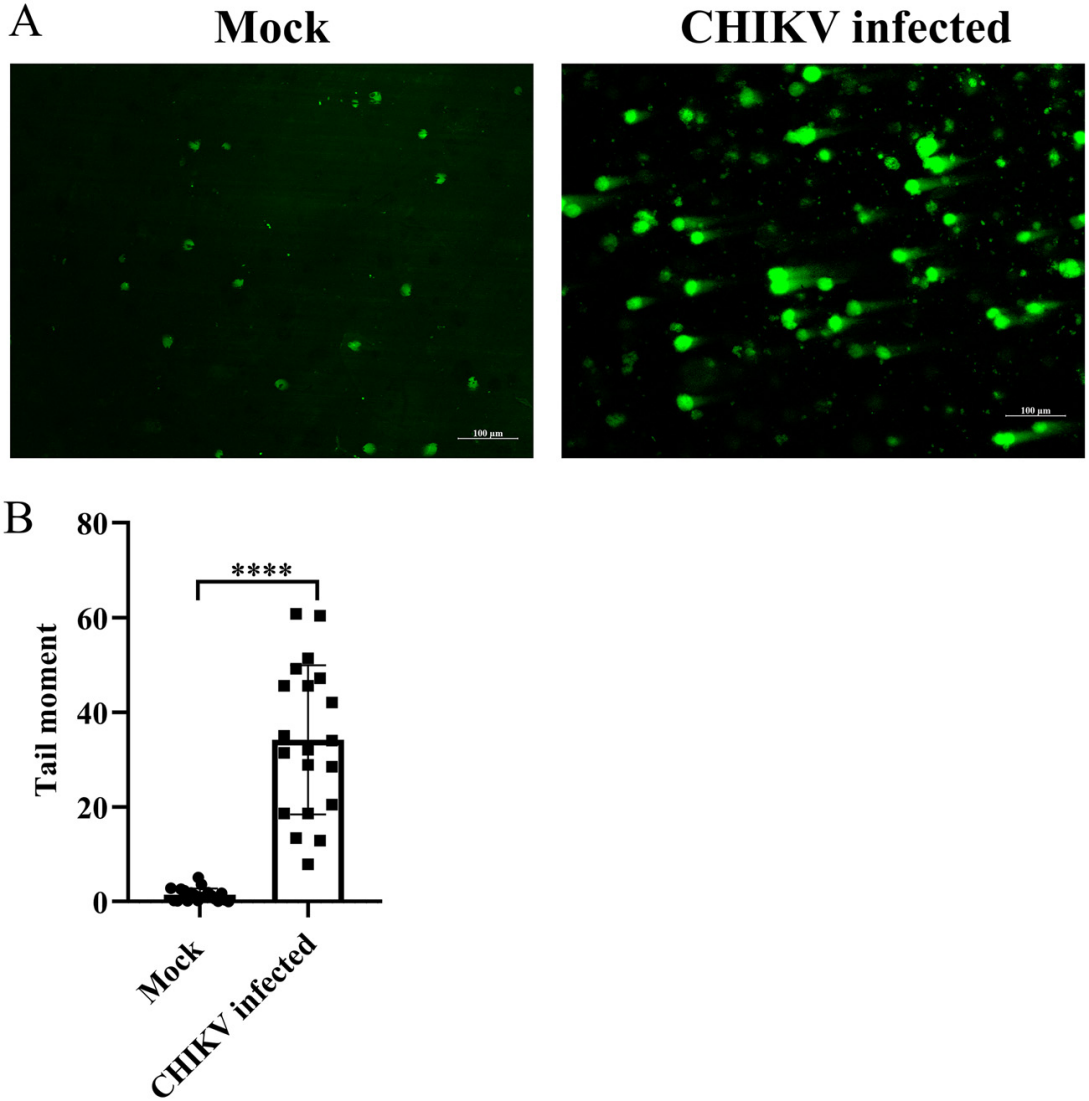
DNA damage is induced by CHIKV infection. The mock and CHIKV-infected Vero cells were harvested at 15 hpi, and the alkaline comet assay was performed. (A) Image depicting the DNA damage in the mock and CHIKV-infected cells. (B) The cluster plot analysis portraying tail moment in the mock and CHIKV-infected samples. Data of three independent experiments are shown as mean ± SD. ****, *P* ≤ 0.0001 was considered statistically significant.

The alkaline comet assay was performed to determine whether DNA damage was induced during CHIKV infection. DNA migration did not occur among most of the control cells, while an increase in the length of DNA migration was observed in CHIKV-infected cells (Fig. 2A and B). The extent of DNA damage in the CHIKV-infected cells was assessed from the tail moment using the Casplab software. CHIKV infection enhanced the degree of DNA damage by 95% compared with the mock cells, indicating a considerable extent of DNA damage (Fig. 2A and B). Taken together, the findings demonstrate that DNA damage is induced upon CHIKV infection.

### Both ATM-ATR pathways facilitate efficient CHIKV infection.

**FIG 3:**
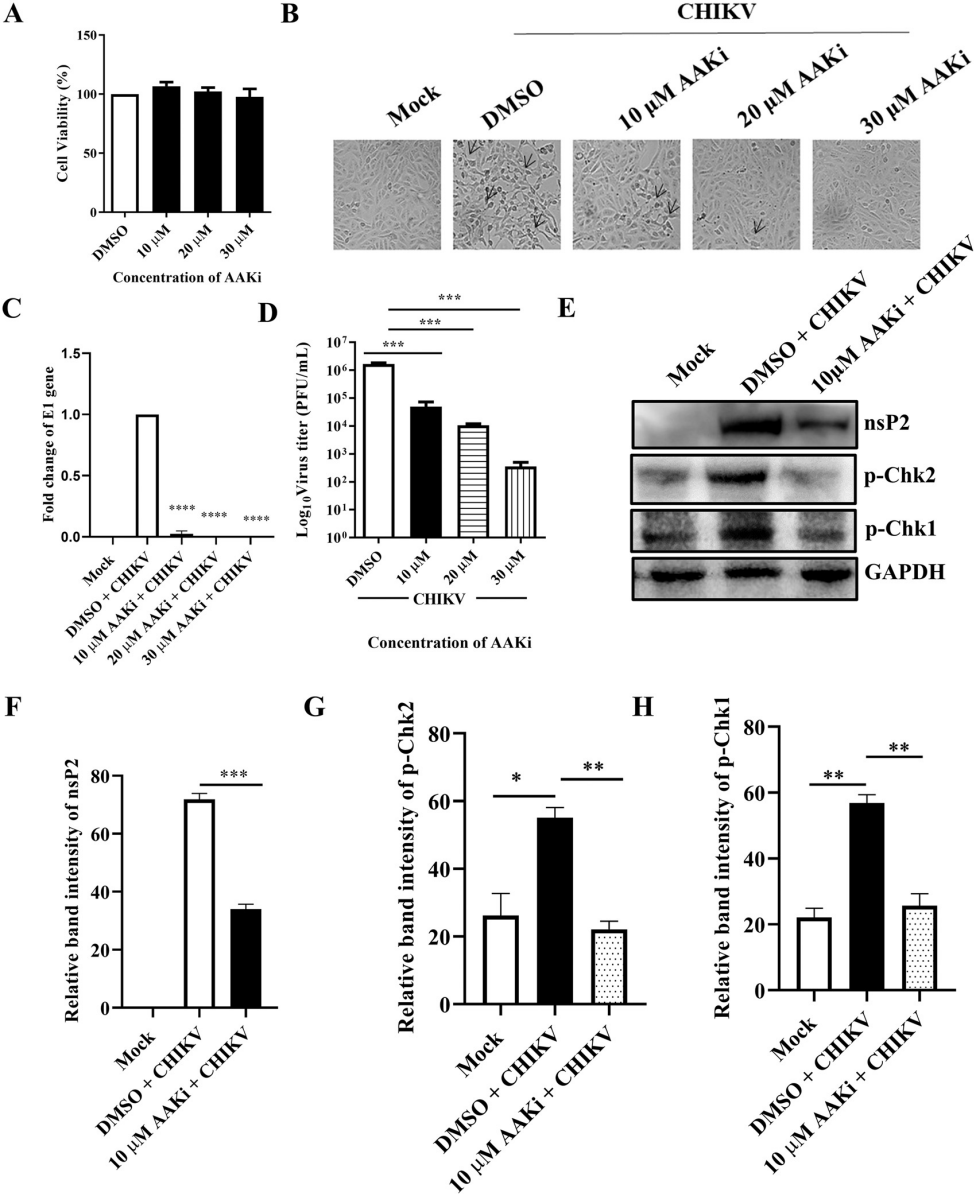
AAKi inhibits CHIKV infection. The Vero cells were treated with different concentrations (10, 20, and 30 μM) of AAKi for 15 h, and the cytotoxicity of the cells was estimated by the MTT assay. (A) Bar diagram showing the viability of cells. (B) Images depicting the CPE of cells which was observed under a microscope (magnification, 20×) with the different concentrations (10, 20, and 30 μM) of drug. (C) Total RNA was isolated from the mock, CHIKV-infected, and drug-treated cells, and the CHIKV-E1 gene was amplified by RT-qPCR. Bar diagram displaying the fold changes of viral RNA. (D) Bar diagram representing the viral titer in the cell supernatant. (E) Western blot image showing the nsP2, p-Chk2, and p-Chk1 protein levels of mock, infected, and infected plus drug-treated cells. GAPDH served as a loading control. (F, G, and H) Bar diagrams depicting the relative band intensities of nsP2, p-Chk2, and p-Chk1. Data of three independent experiments are shown as mean ± SD. *, *P* ≤ 0.05; **, *P* ≤ 0.01; ***, *P* ≤ 0.001; and ****, *P* ≤ 0.0001 were considered statistically significant.

AAKi was used to understand the importance of the DDR pathways in CHIKV infection, and the 3-(4,5-dimethyl-2-thiazolyl)-2,5-diphenyl-2H-tetrazolium bromide (MTT) assay was performed to determine the cytotoxicity of the inhibitor in Vero cells. It was observed that 98% cells were viable at all the concentrations of the drug used (Fig. 3A). After CHIKV infection and drug treatment (10, 20, and 30 μM), cytopathic effect (CPE) was observed under a microscope. Clear reduction in CPE was observed in the drug-treated cells compared with the dimethyl sulfoxide (DMSO) control (Fig. 3B). Next, it was observed that there was more than a 50% reduction in the level of E1 gene expression using Reverse transcription-quantitative PCR (RT-qPCR) (Fig. 3C). Similarly, a plaque assay was performed from the cell culture supernatants, and interestingly, it was observed that the viral titers were also reduced by 70%, 93.7%, and 99.7% in the presence of 10, 20, and 30 μM drug, respectively (Fig. 3D). This finding was further confirmed using Western blot analysis, which revealed a 53.7% reduction in the nsP2 level when 10 μM of the drug was used (Fig. 3E and F). Similarly, the p-Chk2 and p-Chk1 protein levels were also reduced after AAKi treatment (Fig. 3E, G, and H). Collectively, these results indicate that both ATM and ATR pathways are critical for efficient CHIKV infection.

### Efficient inhibition of CHIKV infection by AAKi in mice.

**FIG 4:**
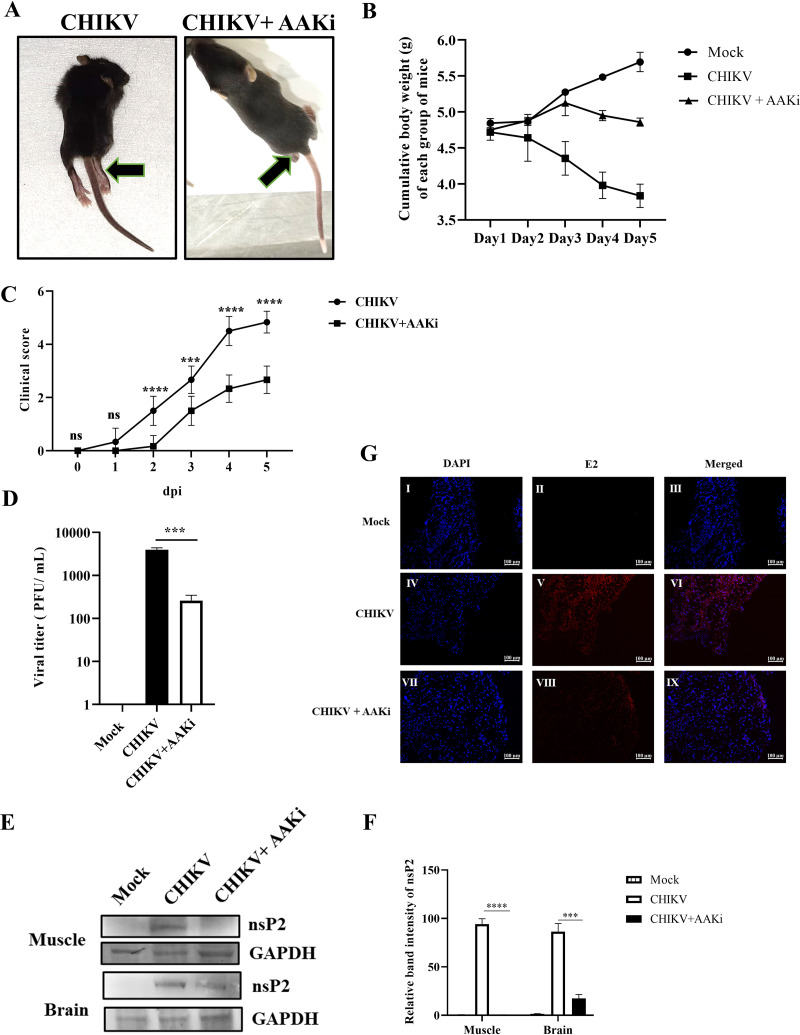
Efficient inhibition of CHIKV infection by AAKi in mice. C57BL/6 mice were infected subcutaneously with 10^7^ PFU of CHIKV and treated with 2 mg/kg of AAKi at 24-h intervals up to 4 dpi. Mice were sacrificed at 5 dpi, and sera and different tissues were collected for further experiments. An equal volume of sera was taken to estimate viral titer by plaque assay. (A) Images of CHIKV-infected and drug-treated mice at 4 dpi, and the arrows show the development of hind limb paralysis in infected mice. (B) Graph exhibiting the cumulative body weight of each group of mice throughout the study. (C) Graph representing the clinical scores of the disease symptoms of mice during CHIKV infection which were monitored from 1 dpi to 5 dpi (*n* = 6). The data are represented as mean ± SD. The 2-way ANOVA with Sidak’s posttest was used. ***, *P* ≤ 0.001; and ****, *P* ≤ 0.0001 were considered statistically significant. (D) Bar diagram showing the viral titers of virus-infected and drug-treated mice sera, obtained through the GraphPad Prism software (*n* = 3) where the one-way ANOVA with Dunnett’s posttest was used. ***, *P* ≤ 0.001 was considered statistically significant. (E) Western blot showing the viral nsP2 protein in muscle and brain tissue samples. GAPDH was used as a loading control. (F) Bar diagram exhibiting the relative band intensities of nsP2 in muscle and brain tissue samples from infected or drug-treated mice, and it was obtained through the GraphPad Prism software (*n* = 3) where the 2-way ANOVA with Sidak’s posttest was used. ***, *P* ≤ 0.001 and ****, *P* ≤ 0.0001 were considered statistically significant. (G) Confocal microscopic images showing the CHIKV-E2-stained muscles from infected or drug-treated mice. Scale bar = 100 μM. Data of three independent experiments are shown as mean ± SD.

To assess the antiviral effect of AAKi *in vivo*, 10- to 12-day-old C57/BL6 mice were infected with CHIKV and treated with 2 mg/kg of body weight of AAKi at every 24-h interval up to 5 days postinfection (dpi). The infected group displayed gradual hind limb paralysis (Fig. 4A) and weight loss (Fig. 4B) and died, while the treated mice showed reduced disease score (Fig. 4C). The plaque assay was performed using the pooled serum samples (from respective groups), and results showed that the viral load was reduced by 93% in AAKi-treated mice compared with that of the control (Fig. 4D). Moreover, Western blot analysis was performed to determine the viral protein level in the mice tissues from the infected and treated groups, which demonstrated 95% and 58% reduction in the nsP2 protein level in the muscle and brain, respectively, in the treated mice (Fig. 4E and F). Furthermore, immunohistochemistry revealed a decrease in E2 level in CHIKV-infected muscle upon AAKi treatment (Fig. 4G). These results indicate that AAKi can protect mice against CHIKV infection.

### Chk2 is crucial for CHIKV infection.

**FIG 5:**
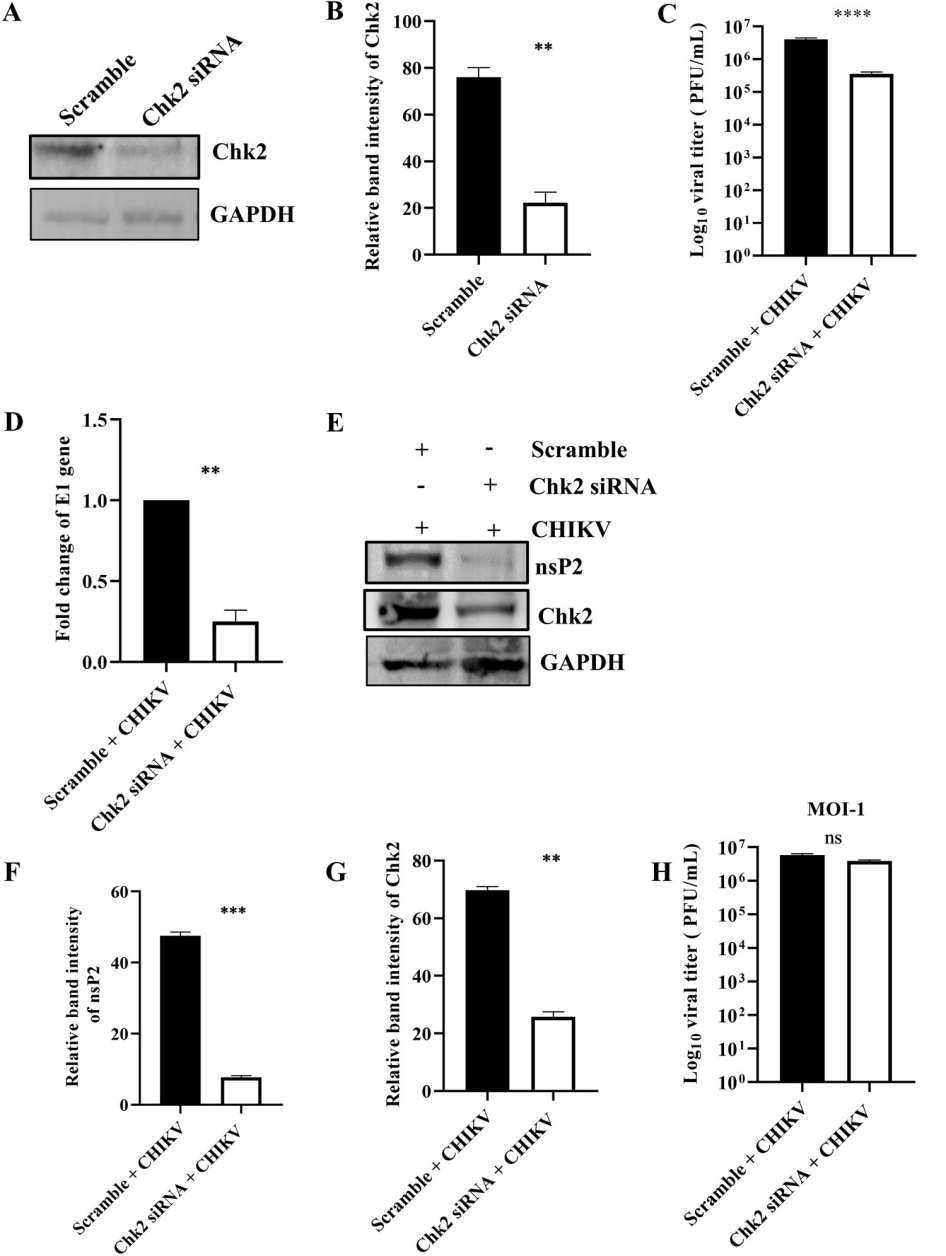
Chk2 is essential for CHIKV infection. The HEK293T cells were transfected with scramble siRNA or 60 pm of Chk2 siRNA. (A) The Chk2 level was estimated by Western blotting, and GAPDH was used as a loading control. (B) Bar diagram showing relative band intensity of Chk2 protein. After 24 hpt, cells were superinfected with CHIKV (MOI, 0.1) and harvested at 15 hpi for further downstream experiments. (C) Bar diagram representing the log_10_ viral titer in the cell supernatant of scramble + CHIKV and Chk2 siRNA + CHIKV samples. (D) Bar diagram showing the fold changes of E1 gene of scramble + CHIKV and Chk2 siRNA + CHIKV samples. (E) Western blot exhibiting the nsP2 and Chk2 protein levels after transfection and superinfection with CHIKV. (F and G) Bar diagrams depicting relative band intensities of the nsP2 and Chk2 proteins. After 24 hpt, cells were superinfected with CHIKV (MOI, 1) and harvested at 15 hpi for plaque assay. (H) Bar diagram representing the log_10_ viral titer in the cell supernatant of scramble + CHIKV and Chk2 siRNA + CHIKV samples. Data of three independent experiments are presented as mean ± SD. Unpaired two-tailed Student’s *t* test was performed for comparing two groups. **, *P* ≤ 0.01; ***, *P* ≤ 0.001; and ****, *P* ≤ 0.0001 were considered statistically significant. ns, not significant.

To investigate the importance of the Chk2 protein for CHIKV infection, 60 pm small interfering RNA (siRNA) was used to silence Chk2 in human embryonic kidney 293T (HEK293T) cells. Cells were harvested at 24 h posttransfection (hpt) and processed for Western blotting to measure the Chk2 level. It was observed that the Chk2 protein level was reduced by 70.7% compared with the scramble siRNA control (Fig. 5A and B). Next, the siRNA-transfected cells were infected with CHIKV (MOI, 0.1), and the supernatants and cell lysates were collected at 15 hpi and processed for plaque assay and Western blotting, respectively. Interestingly, there was 91.2% reduction in viral progeny formation compared with the scramble siRNA control (Fig. 5C). Similarly, there was more than a 50% reduction in E1 expression in RT-qPCR (Fig. 5D), while in Western blot analysis, the nsP2 protein level was found to be reduced by 84% after siRNA transfection (Fig. 5E and F). The Chk2 level was also less, as expected (Fig. 5E and G), and at a higher MOI (MOI, 1), only a 34% reduction in the viral titer was observed after siRNA transfection (Fig. 5H). Together, these results suggest that Chk2 is a host factor crucial for CHIKV infection.

### Chk1 is essential for CHIKV infection.

**FIG 6:**
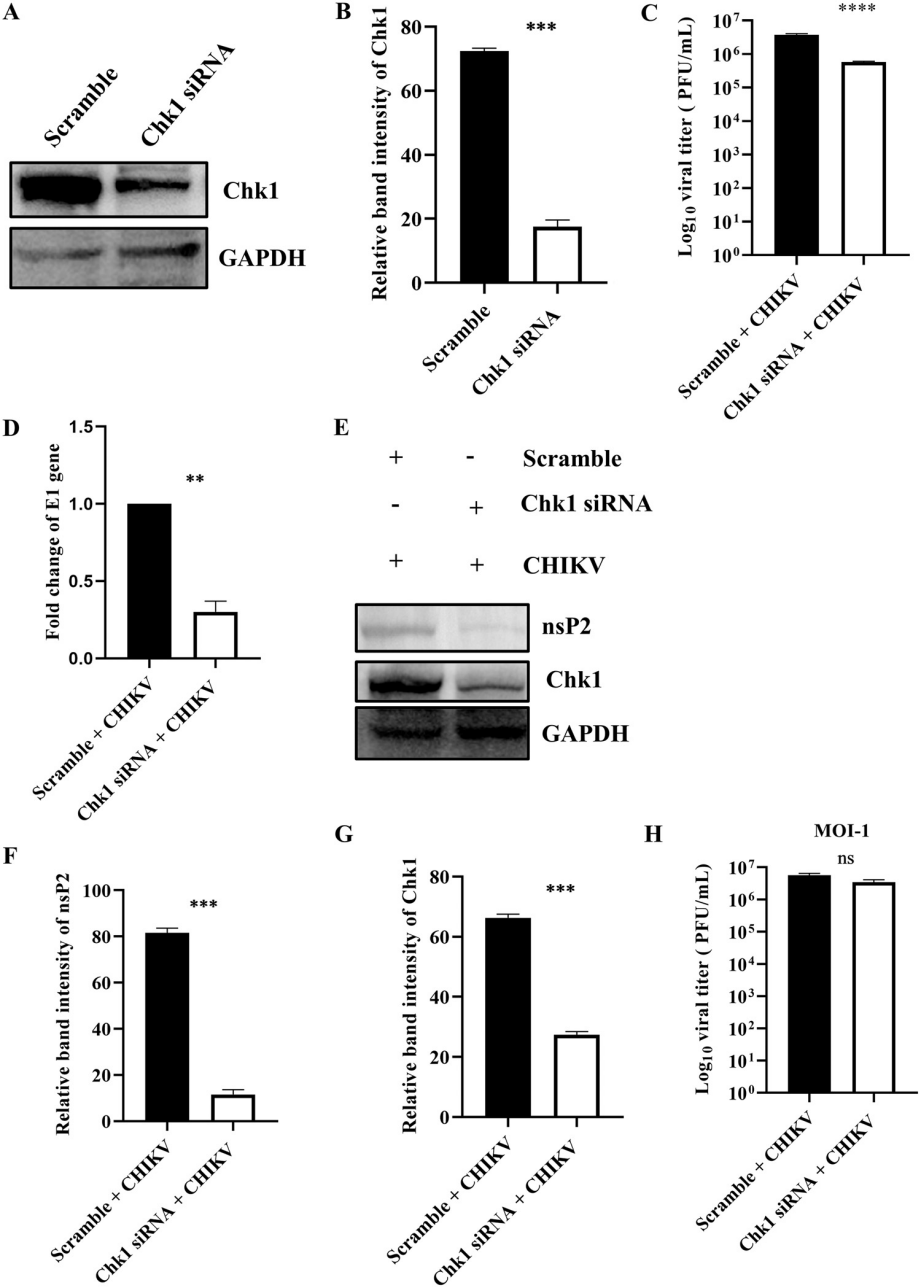
Chk1 is crucial for CHIKV infection. The HEK293T cells were transfected with scramble siRNA or 60 pm of Chk1 siRNA. (A) Chk1 level was assessed by Western blotting, and GAPDH was used as a loading control. (B) Bar diagram displaying relative band intensity of Chk1 protein. After 24 hpt, cells were superinfected with CHIKV (MOI, 0.1) and harvested at 15 hpi for further downstream experiments. (C) Bar diagram representing the viral titer in the cell supernatant of scramble + CHIKV and Chk1 siRNA + CHIKV samples. (D) Bar diagram showing the fold changes of the E1 gene of scramble + CHIKV and Chk1 siRNA + CHIKV samples. (E) Western blot exhibiting the nsP2 and Chk1 protein levels after transfection and superinfection with CHIKV. (F and G) Bar diagrams depicting relative band intensities of the nsP2 and Chk1 proteins. After 24 hpt, cells were superinfected with CHIKV (MOI, 1) and harvested at 15 hpi for plaque assay. (H) Bar diagram representing the viral titer in the cell supernatant of scramble + CHIKV and Chk1 siRNA + CHIKV samples. Data of three independent experiments are represented as mean ± SD. The unpaired two-tailed Student’s *t* test was performed for all the experiments. **, *P* ≤ 0.01; ***, *P* ≤ 0.001; and ****, *P* ≤ 0.0001 were considered statistically significant. ns, not significant.

To understand the role of Chk1 in CHIKV infection, 60 pm siRNA was used to silence Chk1 in HEK293T cells. After 24 hpt, the cells were harvested and processed for Western blotting. It was observed that the Chk1 protein level was reduced by 75.7% after transfecting 60 pm siRNA compared with the scramble siRNA control (Fig. 6A and B). Next, the siRNA-transfected cells were infected with CHIKV (MOI, 0.1), and the supernatants and cell lysates were collected at 15 hpi and processed for plaque assay and Western blotting. Interestingly, it was found to have 85.5% reduction in the viral progeny (Fig. 6C), while more than 50% reduction was observed in the level of the E1 gene in RT-qPCR (Fig. 6D). Moreover, the nsP2 level was reduced by 85.8% after siRNA transfection, as observed in Western blot analysis (Fig. 6E and F). The Chk1 level was also reduced significantly (Fig. 6E and G). When a higher MOI (MOI, 1) was used, the viral titer was decreased by 40% in the siRNA-transfected samples (Fig. 6H). These results suggest that Chk1 is essential for CHIKV infection.

### CHIKV-nsP2 interacts with both Chk1 and Chk2 during CHIKV infection.

**FIG 7:**
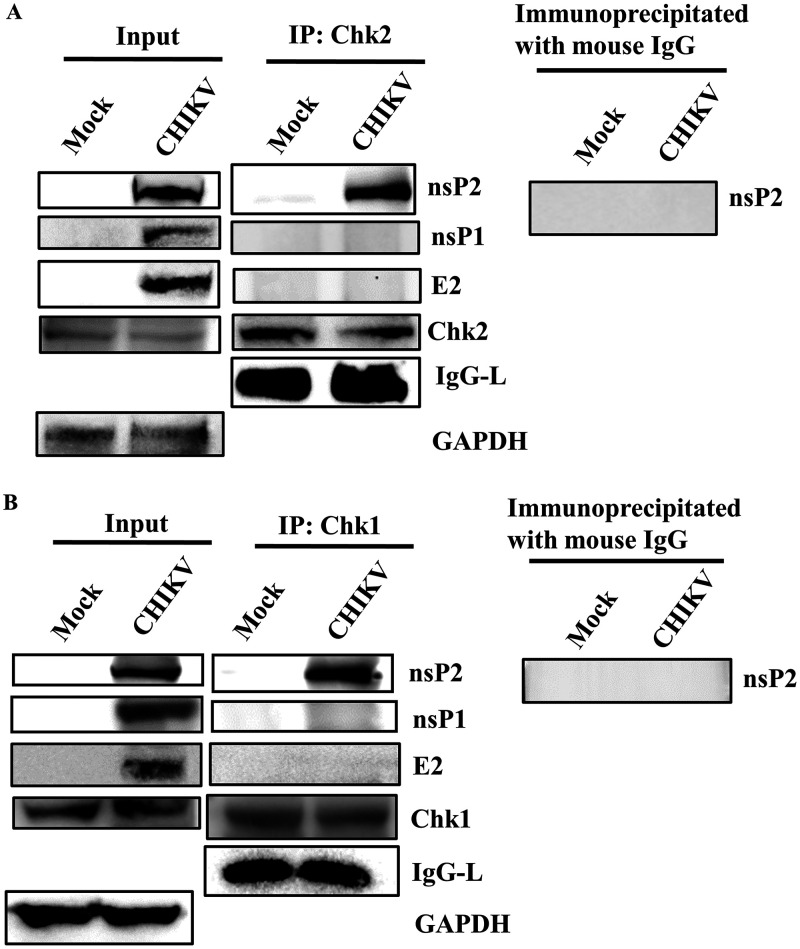
CHIKV-nsP2 interacts with the host Chk2 and Chk1 proteins. The Vero cells were infected with CHIKV and harvested at 6 hpi. The cell lysates were coimmunoprecipitated with the Chk2 and Chk1 antibodies, respectively. (A) Western blot analysis depicting the levels of nsP2, nsP1, E2, Chk2, and GAPDH in the whole-cell lysate (left) where GAPDH was used as a control, and coimmunoprecipitation analysis showing the interaction of the CHIKV-nsP2 and Chk2 proteins (middle). Right panel represents the negative control, where normal mouse IgG was used to immunoprecipitate the protein complex and was probed with the nsP2 antibody. (B) Western blot analysis depicting the levels of nsP2, nsP1, E2, Chk1, and GAPDH in the whole-cell lysate (left) where GAPDH was used as a control, and coimmunoprecipitation analysis showing the interaction of the CHIKV nsP2 and Chk1 proteins (middle). Right panel represents the negative control where normal mouse IgG was used to immunoprecipitate the protein complex and was probed with the nsP2 antibody.

CHIKV infection altered the phosphorylation of the host Chk2 and Chk1 proteins; hence, their association with nsP2 was investigated. CHIKV-infected Vero cells were harvested at 6 hpi and processed for coimmunoprecipitation, followed by Western blotting. It was found that the CHIKV-nsP2 protein was immunoprecipitated by both Chk2 and Chk1. However, nsP1 and E2 do not interact with Chk2 and Chk1. Here, IgG was considered an appropriate negative control and GAPDH was considered a cellular housekeeping gene for assessing the purity of the samples (Fig. 7A and B). Thus, these results suggest that CHIKV-nsP2 interacts with Chk2 and Chk1 during CHIKV infection.

### CHIKV infection leads to both G_1_ and G_2_ arrest.

**FIG 8:**
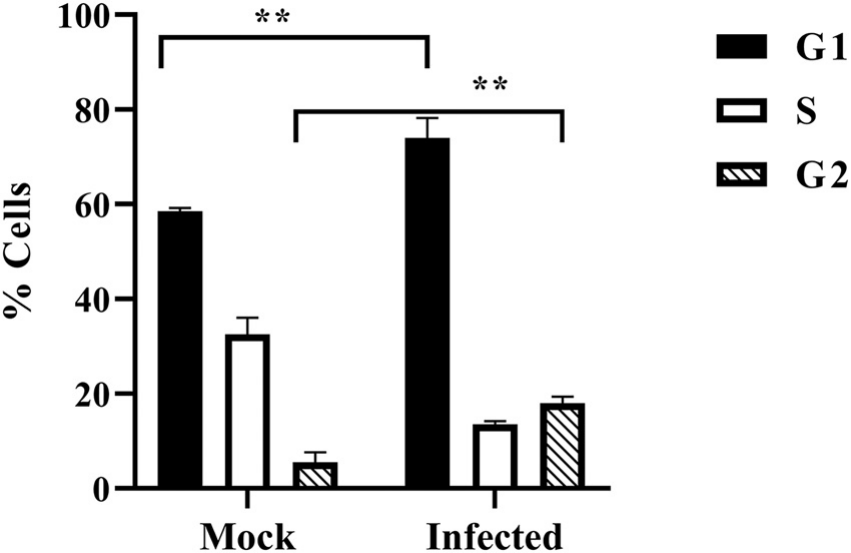
Cells are arrested in G_1_ and G_2_ phases following CHIKV infection. The Vero cells were mock and CHIKV infected and collected at 18 hpi. Cell cycle analysis was performed with propidium iodide (PI) staining. Bar diagram showing quantitation of the data. Data of three independent experiments are shown as mean ± SD. **, *P* ≤ 0.01 was considered statistically significant.

Viruses are known to perturb the cell cycle (19). Here, it was found that both Chk2 and Chk1 were modulated upon CHIKV infection; hence, it was interesting to investigate cell cycle arrest by measuring the DNA content after propidium iodide staining for mock and CHIKV-infected cells at 18 hpi. It was observed that CHIKV infection augmented the percentage of cells in the G_1_ phase from 58.46% to 74.87%, whereas in the G_2_ phase, it was increased from 5% to 18% in the Vero cells (Fig. 8), indicating that CHIKV infection induces cell cycle arrest in both G_1_ and G_2_ phases.

### AAKi abrogates CHIKV infection in the hPBMCs and RAW264.7 cells.

**FIG 9:**
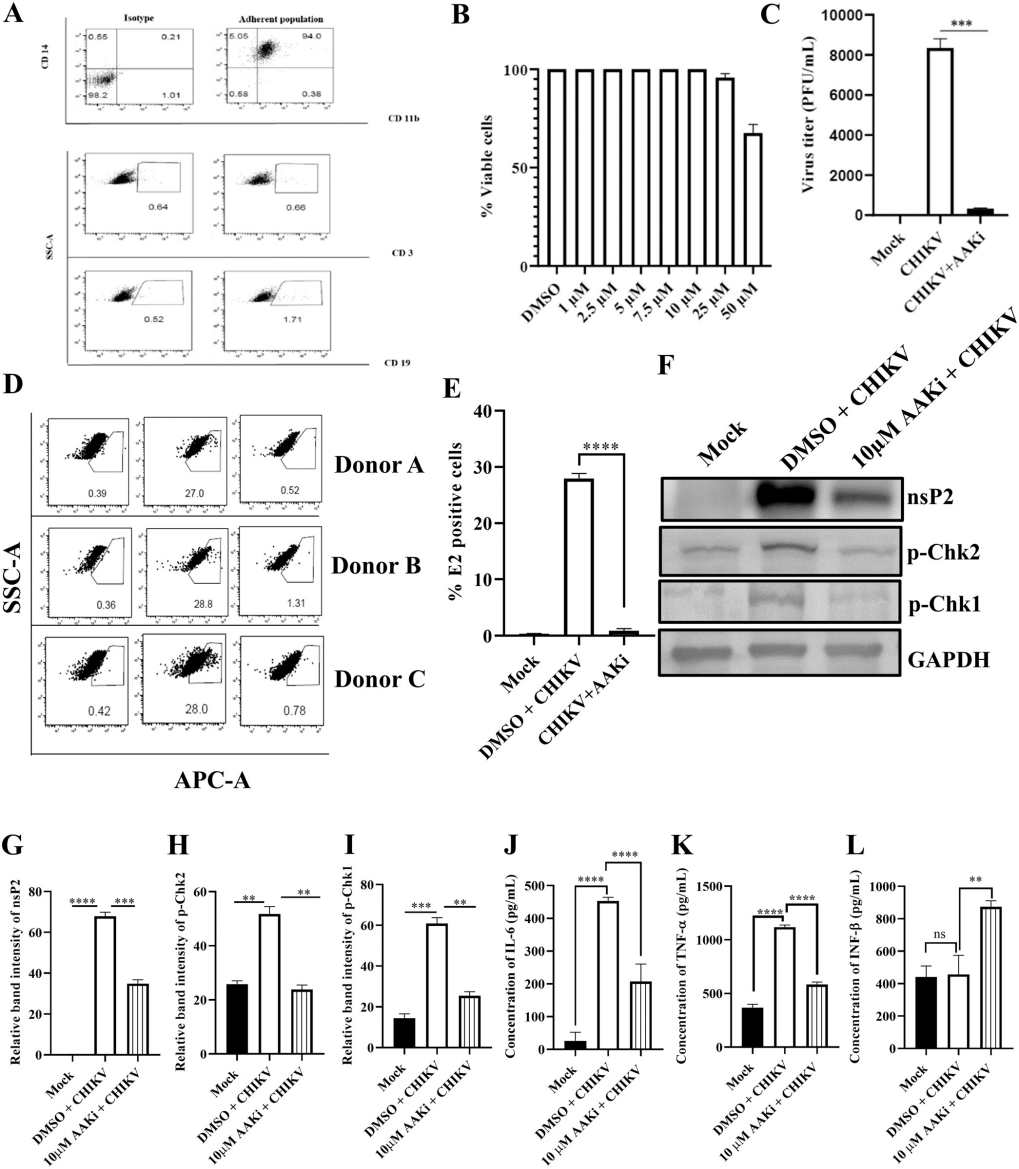
AAKi reduces CHIKV infection in the hPBMC-derived monocyte-macrophage populations *ex vivo* and RAW264.7 cells. The human PBMCs were isolated from blood samples of three healthy donors and infected with CHIKV. (A) Dot plot showing the percentages of B cells (CD19), T cells (CD3), and CD14^+^CD11b^+^ monocyte-macrophage cells from adherent hPBMCs by flow cytometry. (B) Bar diagram representing the cytotoxicity of AAKi in the hPBMC-derived adherent cell populations by the MTT assay. (C) Bar diagram depicting the percentage of the viral particle formation obtained by plaque assay. (D) Dot plot showing the percentage of the viral E2-positive hPBMC-derived monocyte-macrophage population in mock, CHIKV-infected, and AAKi-treated CHIKV-infected samples using flow cytometry. (E) Bar diagram showing the percentage of positive cells for the CHIKV E2 protein, as derived by flow cytometry assay. The RAW264.7 cells were infected with CHIKV and treated with 10 μM AAKi. The cells and supernatants were harvested at 8 hpi for Western blot and sandwich ELISA, respectively. (F) Western blot image depicting the nsP2, p-Chk2, and p-Chk1 protein levels in mock, infected, and infected plus treated cells. GAPDH served as a loading control. (G, H, and I) Bar diagrams representing the relative band intensities of nsP2, p-Chk2, and p-Chk1. (J, K, and L) Bar diagrams showing the levels of secreted cytokines (IL-6, TNF-α, and IFN-β) in the cell culture supernatants. Data of three independent experiments are shown as mean ± SD. **, *P* ≤ 0.01; ***, *P* ≤ 0.001; and ****, *P* ≤ 0.0001 were considered statistically significant. ns, not significant.

Immunological characterization of the hPBMC-derived adherent population was performed using antibodies against the specific markers of the B cells, T cells, and monocyte-macrophage cells in flow cytometry (Fig. 9A). It was found that the adherent population was highly enriched with CD14^+^ CD11b^+^ monocyte-macrophage cells. Then, the adherent population was scraped out and plated in 96-well plates at the density of 5,000 cells per well for the MTT assay. Various concentrations of AAKi (1, 2.5, 5, 7.5, 10, 25, and 50 μM) were added after 24 h of seeding, and the cells were incubated for 24 h at 37°C in the dark. As 95% cells were viable in the presence of 25 μM AAKi (Fig. 9B), all subsequent experiments were performed using 25 μM AAKi. Surprisingly, a 97% reduction was found in the viral progeny formation compared with the DMSO control (Fig. 9C). Next, the effect of AAKi in the hPBMC-derived adherent populations of three healthy donors was investigated upon CHIKV infection *ex vivo*. The CHIKV-infected populations showed 27.93 ± 0.9% E2-positive cells, whereas preincubation with 25 μM AAKi for 3 h led to a decrease in the E2-positive cells up to 0.86 ± 0.4% (Fig. 9D and E). Taken together, the data suggest that AAKi can abrogate CHIKV infection significantly in the human PBMC-derived monocyte-macrophage populations *ex vivo*.

To investigate the activation of innate antiviral responses following CHIKV infection and inhibition of DDR pathways, RAW264.7 cells were infected with CHIKV and treated with 10 μM AAKi. The cells were harvested at 8 hpi, and Western blotting was performed. It was noticed that there was a 48.5% reduction in the nsP2 level (Fig. 9F and G) and a significant decrease in p-Chk2 and p-Chk1 levels after AAKi treatment (Fig. 9F, H, and I). These data suggest that both ATM and ATR pathways enable competent CHIKV infection in the RAW264.7 cells. Additionally, the levels of interleukin-6 (IL-6), tumor necrosis factor alpha (TNF-α), and interferon beta (IFN-β) cytokines were assessed after CHIKV infection and AAKi treatment. It was found that the levels of IL-6 and TNF-α were upregulated, while no significant change was found for IFN-β after CHIKV infection compared with the mock cells. However, the levels of IL-6 and TNF-α were decreased significantly, while the level of IFN-β was elevated following AAKi treatment (Fig. 9J, K, and L). The results indicate that the abrogation of viral infection is because of the inhibition of DDR pathways along with the activation of innate antiviral responses following the blockade of the DDR pathways.

### AAKi downregulates CHIKV infection in the C2C12 cells.

**FIG 10:**
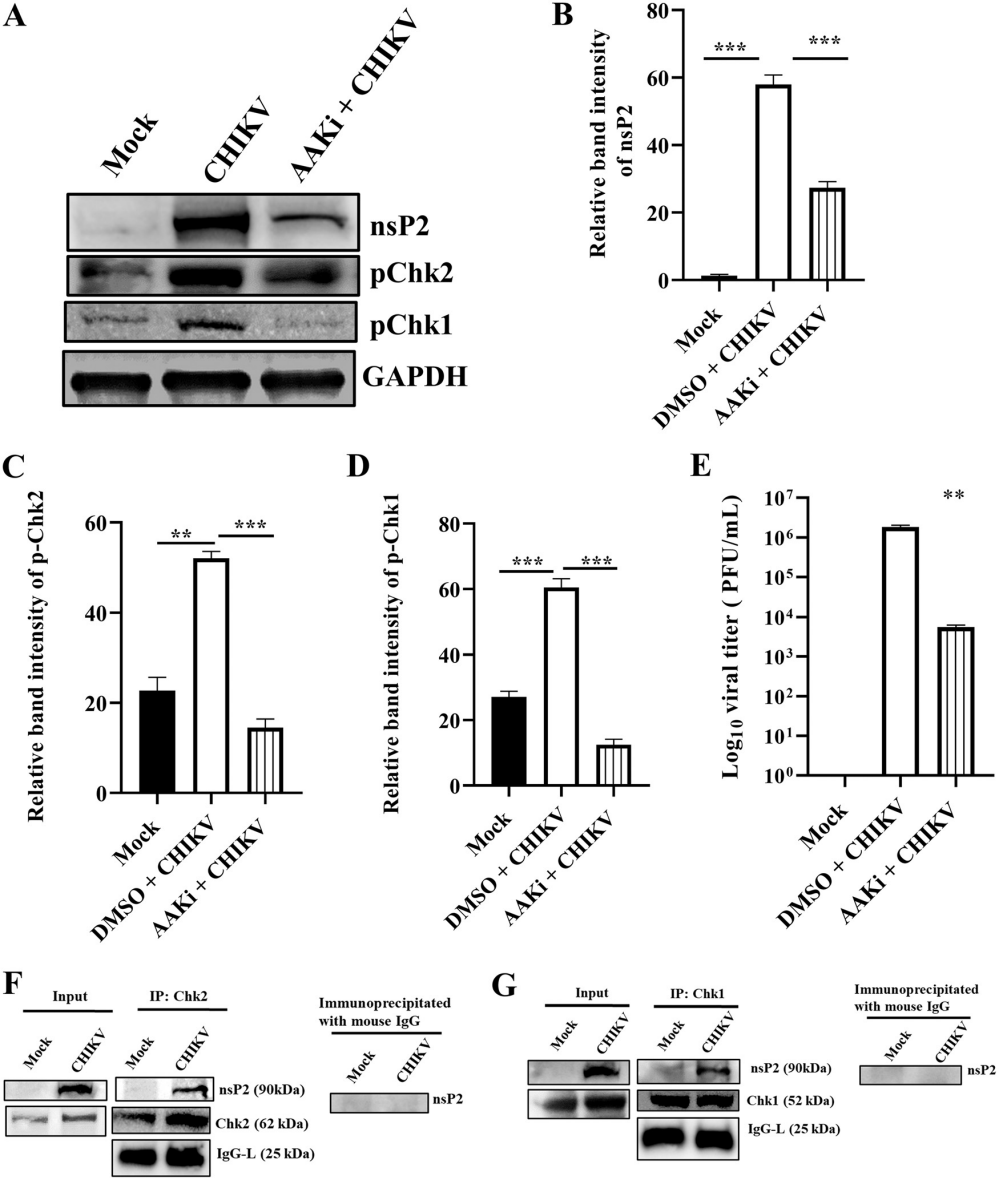
AAKi downregulates CHIKV infection in C2C12 cells. The C2C12 muscle cells were infected and treated with a 10 μM concentration of AAKi, and samples were harvested for Western blot and plaque assay. (A) Western blot image portraying the nsP2, p-Chk2, and p-Chk1 protein levels of mock, infected, and infected plus treated cells. GAPDH served as a loading control. (B, C, and D) Bar diagrams depicting the relative band intensities of nsP2, p-Chk2, and p-Chk1. (E) Bar diagram representing the viral titer in the cell supernatant of the infected and infected plus treated cells. Data of three independent experiments are shown as mean ± SD. **, *P* ≤ 0.01; and ***, *P* ≤ 0.001 were considered statistically significant. The C2C12 muscle cells were infected with CHIKV, and samples were harvested at 6 hpi. The cell lysates were coimmunoprecipitated with the Chk2 and Chk1 antibodies, respectively. (F) Western blot analysis depicting the expressions of nsP2, and Chk2 in the whole-cell lysate (left) and coimmunoprecipitation analysis showing the interaction of the CHIKV-nsP2 and Chk2 protein (middle). Right panel represents the negative control where normal mouse IgG was used to immunoprecipitate the protein complex probed with the nsP2 antibody. (G) Western blot analysis depicting the expressions of nsP2 and Chk1 in the whole-cell lysate (left), and coimmunoprecipitation analysis showing the interaction of the CHIKV-nsP2 and Chk1 proteins (middle). Right panel represents the negative control where normal mouse IgG was used to immunoprecipitate the protein complex and was probed with the nsP2 antibody.

To understand the importance of DDR pathways in CHIKV infection in a physiologically relevant cell, the C2C12 muscle cells were infected with CHIKV and treated with 10 μM AAKi. The cells and supernatants were harvested at 15 hpi and subjected to Western blotting and plaque assay. A reduction of 53% was observed in the nsP2 level (Fig. 10A and B), and p-Chk2 and p-Chk1 levels were reduced significantly after AAKi treatment (Fig. 10A, C, and D). Remarkably, more than a 90% reduction was observed in the viral titer (Fig. 10E). Next, the association of these two host factors with nsP2 was investigated in the CHIKV-infected C2C12 cells. Interestingly, it was found that CHIKV-nsP2 was immunoprecipitated by both Chk2 and Chk1 (Fig. 10F and G). Collectively, these results indicate that both the ATM and ATR pathways are critical for efficient CHIKV infection and that CHIKV-nsP2 interacts with Chk2 and Chk1 during CHIKV infection in the C2C12 cells.

## DISCUSSION

Several DNA and few RNA viruses are known to interact with the cellular proteins involved in the DDR pathways for their efficient infection; however, reports related to alphavirus are scarce. This study is the first one to report that the modulation of the DDR pathways is crucial for effective CHIKV infection.

The study revealed that CHIKV infection activated Chk2 and Chk1 and increased DNA damage by 95%. Inhibition of both the ATM-ATR kinases by AAKi showed that the viral particle formation was reduced by 93.7% in the presence of a 20 μM concentration of the molecule compared with the control. Additionally, the treatment of mice with this drug reduced the disease score substantially. There was a 93% decrease in the viral load, along with diminished viral protein level in AAKi-treated CHIKV-infected C57BL/6 mice. Silencing of Chk2 and Chk1 reduced viral progeny formation by 91.2% and 85.5%, respectively, confirming that both Chk2 and Chk1 are crucial host factors for CHIKV infection. In order to assess the magnitude of inhibition of the Chk2 and Chk1 siRNAs in an MOI-dependent manner, Vero cells were infected with different MOIs (0.1 and 1). The reduction of the viral titer was more with a lower MOI (MOI, 0.1) than that with a higher MOI (MOI, 1) for both the Chk2 and Chk1 siRNAs, respectively, and that finding has also been reported previously (19, 20). Moreover, it was demonstrated that CHIKV-nsP2 interacts with Chk2 and Chk1 during CHIKV infection in the Vero and C2C12 cells. Furthermore, the data indicated that CHIKV infection induces cell cycle arrest in both the G_1_ and G_2_ phases. The CHIKV-infected RAW264.7 cells showed an upregulation of secretory cytokines IL-6 and TNF-α whereas upon AAKi treatment, the levels of both the cytokines were reduced drastically. On the contrary, no significant change was found for IFN-β after CHIKV infection compared with that of the mock cells; however, the level of IFN-β was elevated following AAKi treatment. In addition, the abrogation of CHIKV infection was also established *ex vivo* in the hPBMC-derived monocyte-macrophage populations, RAW264.7, and C2C12 cells using AAKi, confirming the importance of the DDR pathway for an efficient CHIKV life cycle.

In this study, CHIKV was found to activate the proteins associated with DDR pathways, and similar observations have been noted in few other viruses such as bocavirus, human parvovirus, and BK polyomavirus (21–23). Here, it was also observed that CHIKV phosphorylates γH2A.X, which is a hallmark of DDR induction and that has also been reported previously in a case of the Rift Valley fever virus (RVFV) (24). The previous studies have also highlighted the occurrence of DNA damage along with the induction of DDR in few cases like the Zika and Marek’s disease virus infection (25, 26). CHIKV also belongs to the same category, as CHIKV infection induces DNA damage. However, these viral infections (RVFV and BK polyomavirus) do not lead to DNA damage (24, 27). Therefore, the actual mechanistic details underlying the role of these host factors in virus infection is not clear yet and requires further investigation.

Previous findings suggest that the components of the DDR pathways are recruited to the nucleus, while CHIKV replication occurs in the cytoplasm. This information raised a question regarding the modulation of the DDR pathways by the virus to facilitate its replication. Interestingly, the current study exhibited that CHIKV-nsP2 colocalizes with p-Chk2 in the nucleus, and the interaction between Chk1, Chk2, and nsP2 was confirmed using coimmunoprecipitation. Reports have shown that HCV-encoded NS5B interacts with both ATM and CHK2, while its NS3-NS4A interacts with ATM (17). Similarly, vIRF1 of Kaposi’s sarcoma-associated herpesvirus interacts with endogenous ATM (15). ATR and pChk1 were observed to colocalize with the large T antigen (LT) nuclear foci in the Merkel cell polyomavirus (28), indicating that some viral proteins can localize to the nucleus to hijack the host machineries for their benefit.

The results of the *in vitro* experiments showed that AAKi treatment drastically reduced CHIKV infection in the Vero, RAW264.7, and C2C12 cells, which was further corroborated by the observations in the C57BL/6 mice model. While infected mice displayed moderate disease with progressive weight loss, lethargy, hunched posture, ruffled fur, arthritis, and immobility, AAKi (2 mg/kg) mitigated these ailments with a decrease in the viral titer and proteins. The reduction in disease symptoms, nsP2 protein level, and viral titer were well supported by the clinical score. Additionally, the effect of AAKi on CHIKV infection was examined *ex vivo* in the hPBMC-derived monocyte-macrophages. Interestingly, IL-6 and TNF-α levels were found to be elevated after CHIKV infection in the RAW264.7 cell supernatants as observed in a previous study (29), whereas the levels were reduced upon AAKi treatment. In contrast to that result, the IFN-β level was found to be elevated following AAKi treatment compared with the CHIKV-infected cell supernatants, as it was shown in previous study that type I interferons can suppress viral infection (30). It was also reported previously that CHIKV can inhibit IFN production (31, 32). Although CHIKV infection enhanced viral load and the number of E2-positive cells, treatment with AAKi diminished these effects along with an increase in IFN-β level indicating the importance of these pathways in the CHIKV life cycle.

CHIKV infection can control cell cycle arrest in both the G_1_ and G_2_ phases, possibly due to Chk1 and Chk2 activation. Several DNA and RNA viruses have been reported to arrest the cell cycle such that they can create a favorable milieu for viral replication. Cell cycle arrest in the G1 phase has been reported for JEV, influenza A virus, severe acute respiratory syndrome coronavirus (SARS-CoV), Kaposi’s sarcoma-associated herpesvirus, and the murine coronavirus mouse hepatitis virus (MHV), while the G_2_ phase arrest has been known for bocavirus, Epstein-Barr virus, and human immunodeficiency virus (8, 19, 33). Nevertheless, several unresolved questions regarding the precise consequences of virus-induced cell cycle arrest remain, which warrant further investigation.

**FIG 11:**
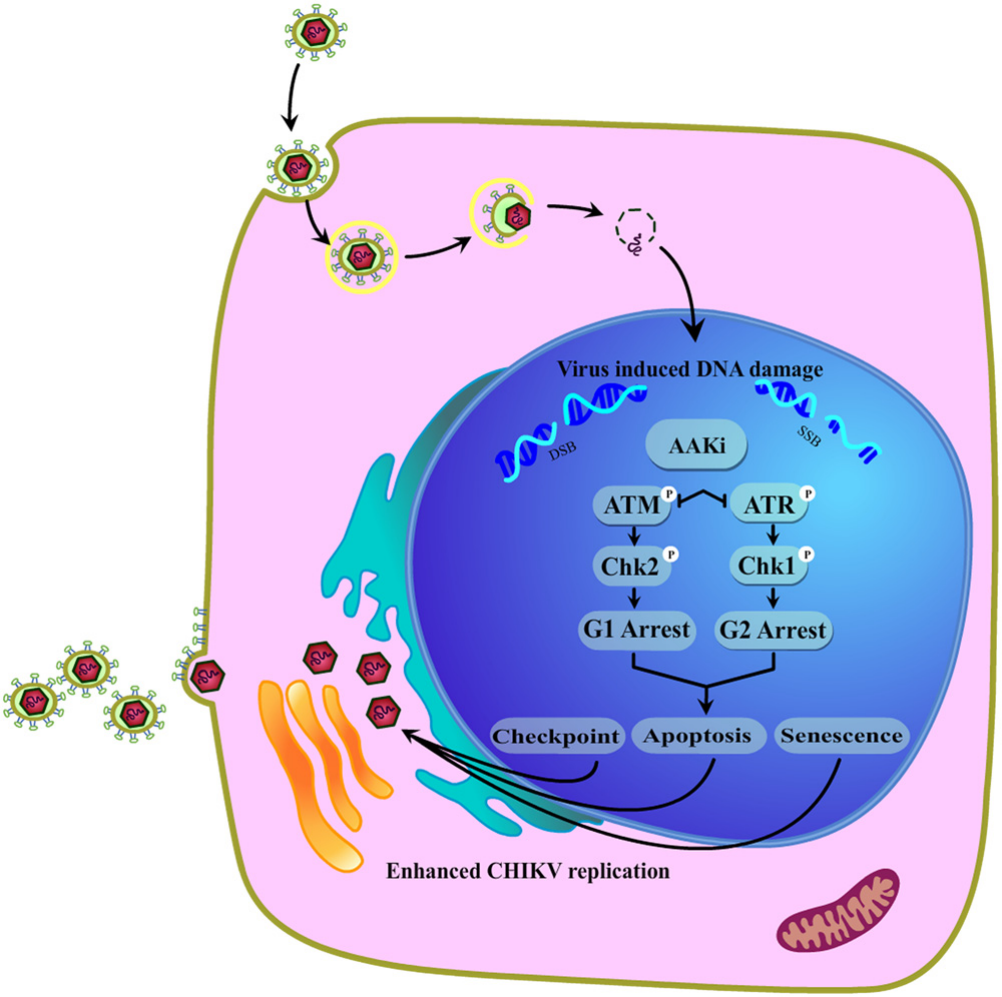
Model portraying the modulation of the DDR pathways by the CHIKV infection. In the current investigation, it was found that CHIKV infection led to extensive DNA damage and stimulated both the ATM/Chk2 and ATR/Chk1 signaling pathways. Inhibition of the crucial host factors linked with DDR pathways caused a drastic reduction in the CHIKV infection. Additionally, it also led to cell cycle arrest in both the G_1_ and G_2_ phases, apoptosis, and senescence that further facilitated viral production.

Hence, CHIKV infection led to extensive DNA damage and stimulated both the ATM/Chk2 and ATR/Chk1 signaling pathways. Following CHIKV infection, Chk2 and Chk1 were phosphorylated, which arrested the cell cycle in G_1_ and G_2_ phases and resulted in apoptosis (20) and senescence (34), which further fostered efficient viral production (Fig. 11). Thus, these two host factors, Chk2 and Chk1, play crucial roles in the CHIKV life cycle.

In conclusion, this is the first study to provide mechanistic insights regarding the induction of the DDR pathways by CHIKV and the interaction of CHIKV-nsP2 with two host factors, Chk2 and Chk1, which are critical for efficient viral infection. In the future, additional investigations will be required to map the amino acids involved in Chk2-nsP2 and Chk1-nsP2 interactions. Furthermore, a mutational study might help to improve our understanding regarding the importance of these interactions in CHIKV infection. AAKi has shown to be a potential inhibitor of CHIKV infection in mice; however, the effect can also be improved further by increasing the dose of the inhibitor to 5 mg/kg of body weight and or by reducing the duration of drug exposure. Overall, the information obtained from this study might contribute to the development of effective therapeutics for the control of the CHIKV infection.

## MATERIALS AND METHODS

### Cells and virus.

The Vero (African green monkey kidney epithelial), human embryonic kidney 293T (HEK293T), and RAW264.7 (mouse monocyte/macrophage) cells were obtained from The National Centre for Cell Science (NCCS), India. The C2C12 (mouse myoblast) cells were a kind gift from Amresh Panda, Institute of Life Sciences, India. Vero, HEK293T, and C2C12 cells were cultured in Dulbecco’s modified Eagle’s medium, (DMEM; PAN Biotech, Germany) supplemented with 10 to 15% fetal bovine serum (FBS) (PAN Biotech) and gentamicin and penicillin-streptomycin (Sigma, USA). The RAW264.7 cells were maintained in RPMI 1640 medium (Gibco RPMI 1640 GlutaMAX; Invitrogen, CA) supplemented with 10% FBS (Gibco FBS; Invitrogen), penicillin-streptomycin, and gentamicin. All the cells were incubated at 37°C in the presence of 5% CO_2_. The Indian CHIKV strain (accession no. EF210157.2) was a gift from M. M. Parida (Defence Research Development Establishment [DRDE], Gwalior, India).

### Antibodies and inhibitors.

The CHIKV-nsP2 and nsP1 antibodies used in this study were developed by our group (35, 36). Chk2, p-Chk2, Chk1, p-Chk1, and γH2A.X (Ser-135) antibodies were procured from Cell Signaling Technologies (Cell Signaling Inc., USA). The E2 antibody was a gift from M. M. Parida (DRDE, Gwalior, India). Glyceraldehyde 3-phosphate dehydrogenase (GAPDH) was purchased from Abgenex, India. The ATM/ATR kinase inhibitor (AAKi) was acquired from Sigma-Aldrich, USA.

### Alkaline comet assay.

Mock and CHIKV-infected cells were harvested at 15 h postinfection (hpi), mixed with low melting agar, and spread onto microscopic slides precoated with normal agarose. Electrophoresis was performed at 25 volts for 20 min using the agarose gel electrophoresis system (Bio-Rad) and processed for the alkaline comet assay as described previously (25). The degree of DNA damage was quantified by investigating randomly selected images in each set (the uninfected and infected groups) using the Casplab software.

### Cytotoxicity assay.

The cytotoxicity of AAKi in Vero cells was determined using the EZcount MTT cell assay kit (Himedia, India) according to the manufacturer’s protocol. Approximately 20,000 cells were plated in a 96-well plate 1 day before the experiment. Upon reaching 80% confluence, the cells were treated with different concentrations of the inhibitor for 15 h, and DMSO was used as the reagent control. The percentage of metabolically active cells was compared with the control cells, and cellular cytotoxicity was determined as described previously (37).

### Viral infection.

The Vero, HEK293T, RAW264.7, and C2C12 cells were infected with different multiplicities of infection (MOIs) of CHIKV according to the protocol described before (37). The cells as well as the supernatants were harvested for various downstream processing.

### Plaque assay.

To calculate viral titer, a plaque assay was performed with the mock, infected, and infected plus drug-treated cell supernatants. After infection, the cells were overlaid with DMEM containing methylcellulose (Sigma, USA) for 4 days, fixed with 8% formaldehyde, and stained with crystal violet as described previously (38).

### Confocal microscopy.

Immunofluorescence analyses were performed as described previously (29). Briefly, the Vero cells were grown on coverslips and infected with CHIKV. At 15 hpi, the cells were fixed with 4% paraformaldehyde and incubated with primary antibodies against viral protein nsP2 (1:1,000) and host protein p-Chk2 (1:750) for 1 h, followed by washing with 1× phosphate-buffered saline (PBS). Next, the cells were incubated with the Alexa Fluor 488 anti-mouse and Alexa Fluor 594 anti-rabbit antibodies for 45 min. Then, antifade reagent (Invitrogen) was used to mount the coverslips to reduce photobleaching. The true point confocal scanning (TCS) SP5 confocal microscope (Leica Microsystems, Germany) was used for acquiring images.

### Animal studies.

Animal experiments were performed in strict compliance with the Committee for the Purpose of Control and Supervision of Experiments on Animals (CPCSEA) of India. All procedures and tests were reviewed and approved by the Institutional Animal Ethics Committee (ILS/IAEC-246-AH/SEPT-21). Mice (10 to 12 days old) were infected subcutaneously with 10^7^ PFU of CHIKV at the flank region of the hind limb, and mock mice were injected with serum-free media as described previously (29). AAKi (2 mg/kg) was administered orally to the treated group of mice (*n* = 3) at every 24-h interval up to 4 days postinfection (dpi). Solvent was provided to mice of the mock and infection-control groups (*n* = 3). For the clinical score studies, all mice (*n* = 6 mice in three groups) were monitored every day up to 5 dpi, and disease outcomes were recorded as no symptoms, 0; fur rise, 1; hunchback, 2; one hind limb paralysis, 3; both hind limb paralysis, 4; and death, 5. At 5 dpi, mice were sacrificed, sera were isolated from blood to assess viral load, and tissues were collected for Western blotting and immunohistochemistry analyses.

### Reverse transcription-quantitative PCR (RT-qPCR).

Cells were harvested and RNA was extracted using TRIzol (Invitrogen, USA). CHIKV-E1 and GAPDH were amplified, and fold changes were calculated as described previously (29).

### siRNA transfection.

Chk2 and Chk1 were silenced using siRNA as described previously (38). In brief, monolayers of HEK293T cells of 70% confluence were seeded in a 6-well plate and were transfected with Lipofectamine 2000 (Thermo Fisher Scientific, USA) using a pool of three different siRNAs corresponding to Chk2 mRNA sequence (sense strand GAAGAGGACUGUCUUAUAAdTdT, CUCAGGAACUCUAUUCUAUdTdT, and GAUCUUCUGUCUGAGGAAAdTdT) and Chk1 mRNA sequence (sense strand GAACCAGUUGAUGUUUGGU dTdT, CUCACAGGGAUAUUAAACC dTdT, and UGUGUGGUACUUUACCAUA dTdT) or using scrambled siRNA as a negative control. At 24 h posttransfection (hpt), the cells were infected with CHIKV with MOI of 0.1 or 1. The supernatants were collected after 15 hpi and subjected to plaque assay, while the cells were harvested for total RNA isolation and Western blot analysis.

### Coimmunoprecipitation.

Mock and CHIKV-infected Vero and C2C12 cells were harvested at 6 hpi followed by lysis with radioimmunoprecipitation assay (RIPA) buffer and subjected to coimmunoprecipitation using the Dynabead protein A immunoprecipitation kit (Thermo Fisher Scientific, USA) as described previously (39).

### Western blotting.

Western blot analysis was performed as described previously (29). Briefly, mock, virus-infected, and drug-treated cells were harvested at different hpi according to the experiments and lysed subsequently with equal volumes of RIPA buffer. Snap-frozen mock, infected, and infected/drug-treated mouse tissues were homogenized using a hand homogenizer and lysed in RIPA buffer using the syringe lysis method. The proteins were separated on a 10% SDS-polyacrylamide gel and transferred onto a polyvinylidene difluoride membrane. The membrane was probed using antibodies as recommended by the manufacturer. The blots were developed using the Immobilon Western chemiluminescent horseradish peroxidase (HRP) substrate (Millipore, USA). The Image J software was used for the quantification of protein bands from three independent experiments.

### Immunohistochemistry.

For immunohistochemistry, formalin-fixed tissue samples were dehydrated and embedded in paraffin wax, and serial paraffin sections of 5-μm thickness were obtained as described previously (29). Briefly, the tissue sections were incubated with the primary E2 antibody followed by the secondary Alexa Fluor 594 antibody (anti-mouse; Invitrogen, USA). The slides were mounted with 4′,6-diamidino-2-phenylindole (DAPI; Invitrogen), and coverslips were applied to the slides.

### Cell cycle analysis.

The mock and infected cells were washed, harvested, and resuspended in chilled 70% ethanol for fixation. Next, they were stained with propidium iodide staining solution containing RNase A, and cell cycle analysis was performed using an LSRFortessa instrument as described previously (19).

### Isolation of human peripheral blood mononuclear cells (hPBMCs) and CHIKV infection.

Human PBMCs were isolated from the blood samples of three healthy donors as described previously (29). The studies involving human participants were reviewed and approved by the Institutional Human Ethics Committee, Institute of Life Sciences. The Institutional Ethics Committee (IEC)/Institutional Review Board (IRB) reference number is 96/HEC/2020. Written informed consent was obtained from the participants’ legal guardian/next of kin. All the adherent cells were detached after 5 days. The MTT assay was performed with various concentrations of AAKi for the adherent hPBMCs. After 1 d, adherent cells were pretreated with 25 μM AAKi for 2 h and subjected to CHIKV infection at an MOI of 5 for 2 h. The infected cells were harvested at 12 hpi, followed by fixation with 4% paraformaldehyde. Next, the cells were subjected to intracellular staining to detect the viral protein E2 and surface staining for immunophenotyping of the adherent population using flow cytometry. For immunophenotyping, the fluorochrome-conjugated anti-human CD3, CD11b, CD14, and CD19 antibodies (Abgenex, India) were used as described previously (29).

### Sandwich enzyme-linked immunosorbent assay (ELISA).

The RAW264.7 cells were infected with CHIKV and treated with 10 μM AAKi. The culture supernatants were subjected to the sandwich ELISA (BD OptEIATM sandwich ELISA kit, USA) to estimate the tumor necrosis factor-alpha (TNF-α) and interleukin-6 (IL-6) levels according to a protocol described previously (29). The mouse IFN beta SimpleStep ELISA kit (Abcam, UK) was used to estimate the interferon-β (IFN-β) level according to the manufacturer’s instruction. The concentrations of cytokines were determined from the standard curves plotted using known concentrations of corresponding recombinant cytokines. The cytokine concentrations were determined at 450 nm using the Epoch 2 (BioTek, USA) microplate reader.

### Statistical analysis.

The GraphPad Prism version 8.0.1 software was used for statistical analyses. Data of three independent experiments are shown as mean ± SD. *, *P* < 0.05; **, *P* < 0.01; ***, *P* < 0.001; and ****, *P* < 0.0001 were considered statistically significant. An unpaired two-tailed Student’s *t* test was performed for comparing two group. For three or more groups, the one-way or two-way analysis of variance (ANOVA) with Dunnett’s and Sidak’s posttests were used.

### Data availability.

The data that support the findings of this study are available from the corresponding author upon reasonable request. Some data may not be made available because of privacy or ethical restrictions.
